# XIAP Is a Predictor of Cisplatin-Based Chemotherapy Response and Prognosis for Patients with Advanced Head and Neck Cancer

**DOI:** 10.1371/journal.pone.0031601

**Published:** 2012-03-05

**Authors:** Xi-Hu Yang, Zhi-En Feng, Ming Yan, Sayaka Hanada, Hui Zuo, Cheng-Zhe Yang, Ze-Guan Han, Wei Guo, Wan-Tao Chen, Ping Zhang

**Affiliations:** 1 Department of Oral and Maxillofacial Surgery, Ninth People's Hospital, Shanghai Jiao Tong University School of Medicine, Shanghai, China; 2 Shanghai Key Laboratory of Stomatology, Shanghai, China; 3 Department of Oncology and Diagnostic Sciences, University of Maryland Dental School, Baltimore, Maryland, United States of America; Karolinska Institutet, Sweden

## Abstract

**Background:**

Approximately 60–80% of patients with advanced head and neck squamous cell carcinoma (HNSCC) die within five years after diagnosis. Cisplatin-based chemotherapy is the most commonly used palliative treatment for these patients. To evaluate the prognostic value of X-linked inhibitor of apoptosis (XIAP) level as a potential biomarker in these patients, we investigated the relationship between XIAP expression and cisplatin response of these patients and their prognosis.

**Methodology/Principal Findings:**

Sixty patients with advanced HNSCC were recruited in this study. Expression of XIAP was examined both before and after chemotherapy and was correlated with chemotherapy response, clinicopathology parameters and clinical outcomes of the patients. We found that XIAP was expressed in 17 (20.83%) of the 60 advanced HNSCC samples and the expression was significantly associated with cisplatin resistance (P = 0.036) and poor clinical outcome (P = 0.025). Cisplatin-based chemotherapy induced XIAP expression in those post-chemotherapy samples (P = 0.011), was further associated with poorer clinical outcome (P = 0.029). Multivariate analysis demonstrated that only alcohol consumption, lymph node metastasis and XIAP level were independently associated with the prognosis of advanced HNSCC patients. Inhibiting XIAP expression with siRNA in XIAP overexpressed HNSCC cells remarkably increased their sensitivity to cisplatin treatment to nearly a 3 fold difference.

**Conclusions/Significance:**

Our results demonstrate that XIAP overexpression plays an important role in the disease course and cisplatin-resistance of advanced HNSCC. XIAP is a valuable predictor of cisplatin-response and prognosis for patients with advanced head and neck cancer. Down-regulation of XIAP might be a promising adjuvant therapy for those patients of advanced HNSCC.

## Introduction

Head and neck squamous cell carcinoma (HNSCC) is the fifth most common cancer worldwide and is the most common neoplasm in central Asia [1]. Although early-stage HNSCC have high cure rates, up to 50% of patients present with advanced disease [2]. Among these advanced stage HNSCC patients, 60–80% will die within 5 years after diagnosis [3]. Currently, cisplatin-based chemotherapy is the most commonly used palliative treatment for these patients. However, in clinic only a limited number of patients benefit from cisplatin-based chemotherapy; other patients are resistant to this therapy and some will die due to treatment-related toxicity [4]. Therefore, it is essential to look for predictors or potential biomarkers that may help to identify the patients who may benefit from cisplatin-based chemotherapy.

Inhibitors of apoptosis proteins (IAPs) represent one set of potent endogenous modulators of apoptosis in mammalian cells, which consist of eight members: XIAP, cIAP1, cIAP2, survivin, NIAP, Bruce, ML-IAP and ILP-2 [5]. These proteins mediate multiple biological functions that include binding to and inhibiting caspases, regulating cell cycle progression, and modulating receptor-mediated signal transduction [6]. Among them, X-linked inhibitor of apoptosis (XIAP) is one of the most potent inhibitor of caspases and apoptosis. XIAP can directly bind to and inhibit both the initiator and effector caspases and inhibit both mitochondrial-dependent and -independent apoptotic pathways [7], [8], [9]. Recent findings have shown *in vitro*, XIAP can cause resistance among tumor cells when exposed to a variety of apoptotic stimuli, including chemotherapy [10], [11]. However, it is not known if XIAP expression level could be used to predict the cisplatin response of advanced HNSCC. Therefore, the aim of our study was to investigate the expression of XIAP in advanced HNSCC and its relationship with cisplatin response and prognosis of these patients.

## Results

### XIAP expression level is associated with a poor clinical outcome of advanced HNSCC patients

**Figure 1:**
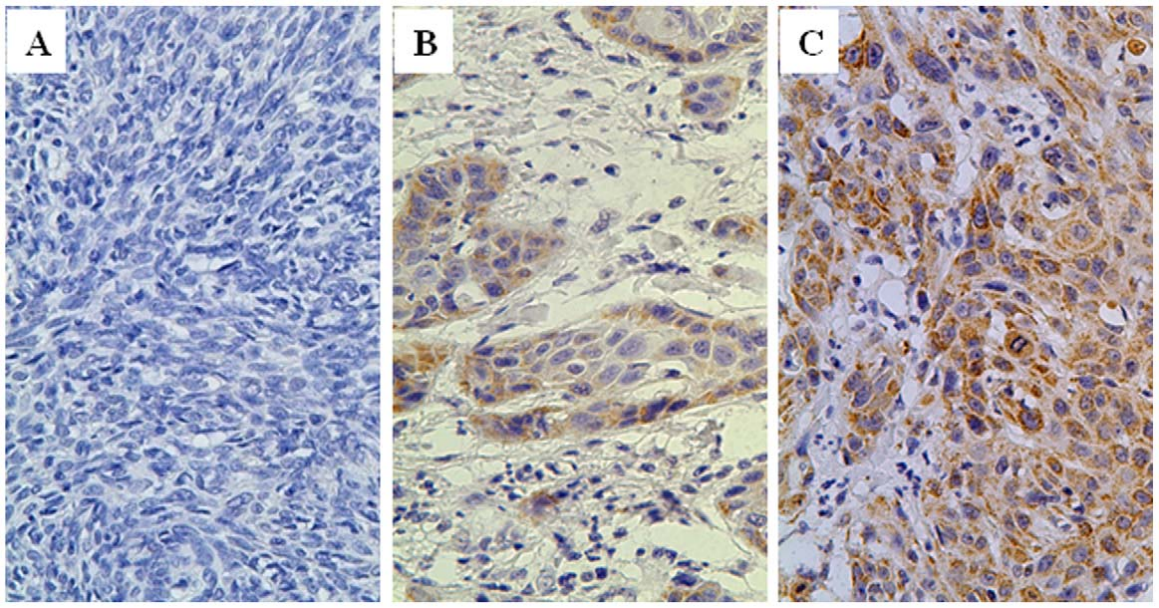
Immunohistochemical staining of XIAP in advanced HNSCC (×400). A: Negative control with PBS instead of first antibody; B: Low expression of XIAP(the percentage of positive rate <25%); C: High expression of XIAP(the percentage of positive rate >25%).

**Figure 2:**
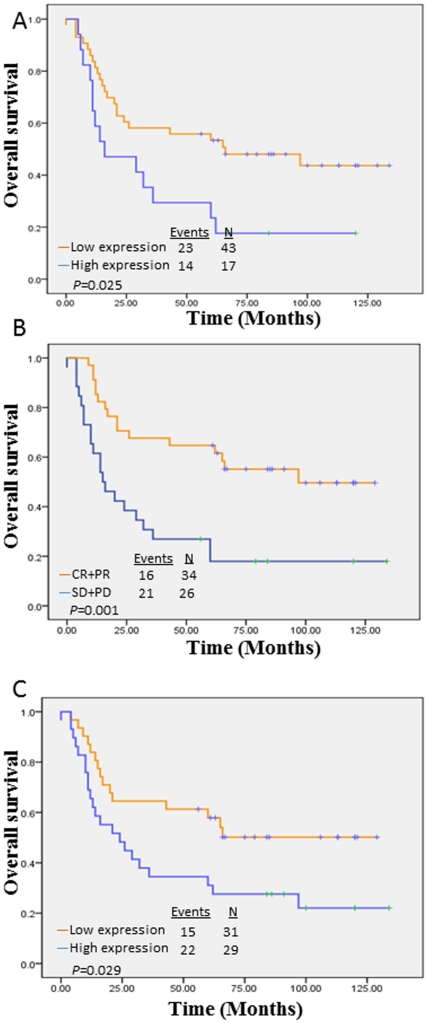
Overall survival. A: Overall survival rate by XIAP scores. Patients whose tumors expressed high levels of XIAP generally had a poorer prognosis than those patients whose tumors expressed low levels of XIAP in pre-chemotherapy cancer tissue; B: Overall survival rate by chemoresponse. Patients whose tumors were responsive to chemotherapy generally had a better prognosis than those patients whose tumor was resistant to chemotherapy; C: XIAP scores of post-chemotherapy samples. XIAP levels in post-chemotherapy samples were also significantly related to the patient overall survival rates.

XIAP was mainly localized in the cytoplasm of tumor cells (**Figure 1**), with highly variable positive rate from 1%–85%. Nucleus staining was occasionally observed in some post-chemotherapy samples. Patients whose tumors expressed high levels of XIAP generally had a poorer prognosis than those patients whose tumors expressed low levels of XIAP in pre-chemotherapy's cancer tissue (overall survival *P* = 0.025, Log Rank test, **Figure 2**). XIAP expression rate was not correlated with the TNM stage, pathologic grade, smoking and alcohol history of these patients with advanced HNSCC (**Table 1**). In multivariate analysis, lymph node metastasis, alcohol consumption and XIAP expression (pre-chemotherapy) were independent risk factors for patients' prognosis (**Table 2**).

**Table 1:** The correlations between XIAP expression and patient characteristics.

Characteristics	pre-chemotherapy			post-chemotherapy		
	Low	High	*P*	Low	high	*P*
Gender						
Male	31	8	0.067	20	19	0.935
Female	12	9		11	10	
Age						
<60	26	11	0.761	16	21	0.098
≥60	17	6		15	8	
cTNM stage						
III	13	8	0.218	9	12	0.316
IV	30	9		22	17	
Pathologic grade						
I	22	9	0.311	13	18	0.465
II	19	6		17	8	
III	2	2		1	3	
Lymph node status						
Positive	25	13	0.184	15	23	
Negative	18	4		16	6	
Chemoresponse						
SD+PD	15	11	0.036	8	18	0.005
PR+CR	28	6		23	11	
Smoking history						
Smoking	20	7	0.708	14	13	0.979
No smoking	23	10		17	16	
Alcohol history						
Drinker	18	7	0.961	11	14	0.315
Nondrinker	25	10		20	15	
Overall survival						
Censored	20	3	0.025	16	7	0.029
Event	23	14		15	22	

**Table 2:** Cox proportional hazards regression models in estimating overall survival.

Characteristics	Hazard ratio	95% Confidence interval	*P*
**Univariate survival analysis**			
Age	1.254	0.653–2.405	0.497
Clinical stage	0.885	0.455–1.722	0.719
Pathologic grade	1.303	0.669–2.536	0.437
Gender	0.828	0.415–1.653	0.593
Lymph node status	2.772	1.301–5.906	0.008
XIAP expression(pre-chemotherapy)	2.108	1.077–4.123	0.029
XIAP expression(post-chemotherapy)	2.045	1.059–3.950	0.033
Smoking history	0.635	0.333–1.211	0.168
Drinking history	0.442	0.231–0.847	0.014
**Multivariate survival analysis**			
Lymph node status	2.544	1.186–5.457	0.016
Alcohol history	0.398	0.202–0.783	0.008
XIAP expression(pre-chemotherapy)	2.311	1.151–4.643	0.019

### XIAP expression level was associated with chemotherapy response of patients with advanced HNSCC

All of these advanced HNSCC patients had finished one cycle of chemotherapy. Among them, 34 cases were complete response (CR) and partial response (PR) to chemotherapy and 26 cases were progressive disease (PD) and stable disease (SD). The patients whose tumors expressed high levels of XIAP were significantly more resistant to cisplatin chemotherapy and generally had poorer chemotherapy responses (*P* = 0.005, **Table 1**). XIAP expression levels were greatly increased in the post-chemotherapy HNSCC tissues compared with the pre-chemotherapy samples (*P* = 0.011, **Table 3**). XIAP levels in post-chemotherapy samples were also significantly related to the overall survival rates of these patients (*P* = 0.029) (**Figure 2**), although in multivariate analysis, it was not an independent factor related to the patients' outcomes.

**Table 3:** The expression of XIAP in pre- and post- chemotherapy samples.

XIAP expression	Low (%)	High (%)
Pre-chemotherapy	43(72)	17(28)
Post-chemotherapy	31(52)	29(48)
*P*	0.011	

### Inhibiting XIAP expression sensitized HNSCC cell lines to cisplatin treatment

**Figure 3:**
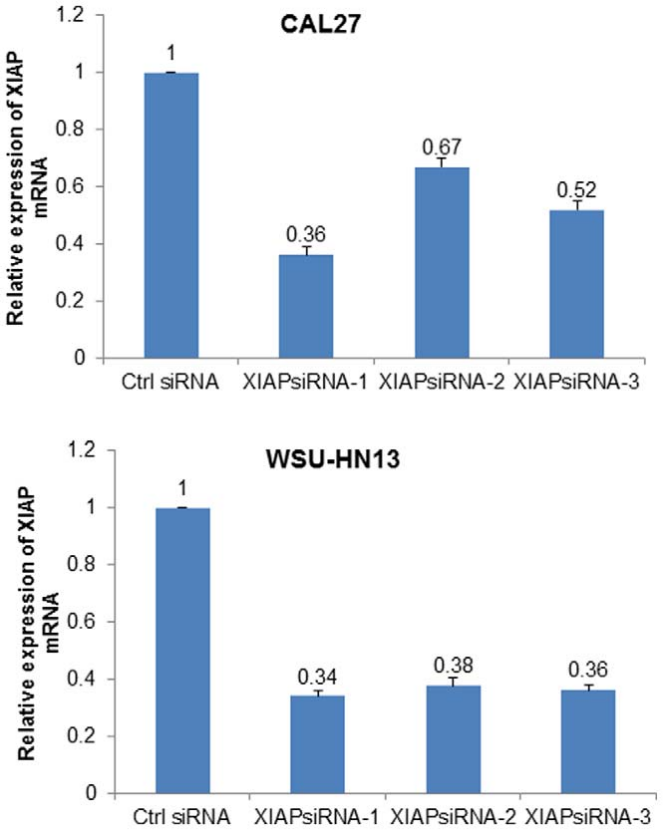
XIAP expression inhibited by siRNA. Inhibition efficacy of siRNA-1, siRNA-2 and siRNA-3 on the expression of XIAP mRNA was examined in CAL27 cell (Upper) and WSU-HN13 cell (Lower) with Real-time PCR. XIAP siRNA1 treatment group obtained near 70% reduction of XIAP mRNA expression in both cells.

**Figure 4:**
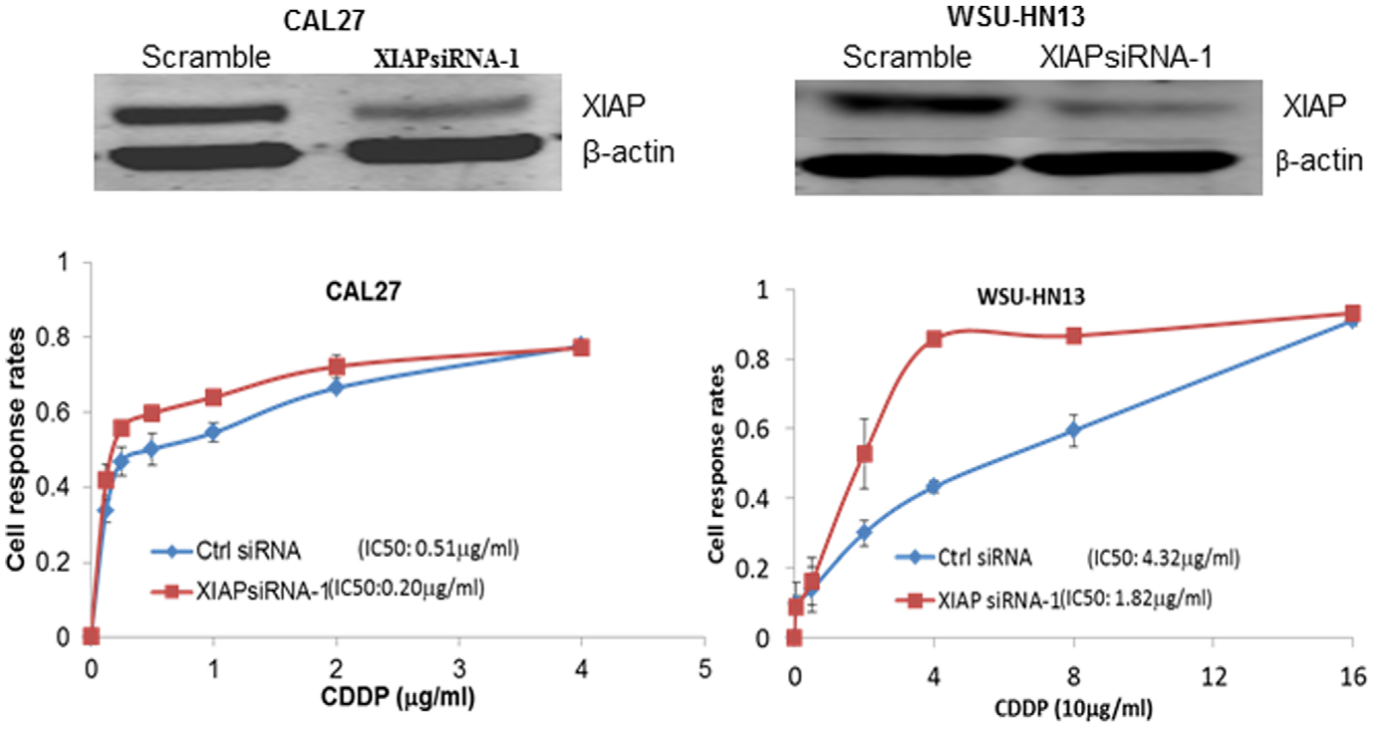
Inhibiting XIAP expression sensitized both CAL27and WSU-HN13 to cisplatin treatment. XIAP siRNA-1 effectively inhibited the expression of XIAP protein in both CAL27 and WSU-HN13 cells (Upper) and decreased the cisplatin IC50 value from 0.51 µg/ml to 0.20 µg/ml in CAL27 and from 4.32 to 1.82 µg/ml in WSU-HN13 (Lower).

To investigate the casual relationship of XIAP expression and drug response of patients, we used siRNA to inhibit XIAP expression in HNSCC cell line CAL27 and WSU-HN13. Three siRNAs were designed to inhibit the expression of XIAP in CAL27 and WSU-HN13 cells, and among them, siRNA1 treatment group obtained near70% reduction of XIAP mRNA expression in both cells (**Figure 3**). Compared with the negative control group, cisplatin IC50 value in siRNA1 group decreased from 0.51 µg/ml to 0.20 µg/ml (*P* = 0.05, **Figure 4**) in CAL27 and from 4.32 to 1.82 µg/ml in WSU-HN13.

## Discussion

Cisplatin-based neoadjuvant chemotherapy, surgery and radiation therapy used to be regarded as standard regime for advanced HNSCC patients in high risk; however, the therapeutic effects of neoadjuvant therapy remain inconclusive due to many randomized trials failing to show a survival advantage with the use of neoadjuvant chemotherapy [12], [13]. Although indiscriminate administration of neoadjuvant chemotherapy addresses no benefit to patients' outcome (five-year survival rate was 38.33% in our study), we found those patients who achieved a clinical response had a more favorable prognosis (p = 0.001, log-rank test), which is consistent to some previous reports [12], [13]. From this point of view, evaluating how to selectively choose patients who would positively benefit from neoadjuvant chemotherapy was the decisive factor for successful treatment [4].

In this study, we found a strong relationship between the expression level of XIAP and the clinical response and prognosis of patients with advanced HNSCC. Low XIAP expression was closely correlated with chemotherapy response and favorable prognosis, whereas high XIAP expression may predict chemotherapy failure and poor outcome. The results are consistent with previous reports showing that the down-regulation of XIAP sensitizes cancer cells to therapeutic drugs in lung cancer, prostate cancer, ovarian cancer and pancreatic cancer[14], [15], [16], [17], [18], [19], [20]. Importantly, we found that cisplatin-based chemotherapy greatly induced the expression of XIAP in advanced HNSCC. The higher XIAP level in post-chemotherapy samples also associated with a poorer prognosis of patients. Our results show that XIAP expression is a primary cause of treatment failure and chemotherapy-induced XIAP expression led to a poor prognosis of those drug-resistant patients. Pre-selected XIAP negative patients may benefit from cisplatin-based neoadjuvant treatment. Our *in vitro* data further proposed a potential value of inhibiting XIAP expression to enhance the effectiveness of chemotherapy.

It should be noted that contrary to most studies, we observed a positive association between alcohol consumption and overall survival of advanced HNSCC patients in this study. Interesting, a similar result has been published recently, which proposed the differences of our data and cultural tradition might be caused by different drinking habits: the advanced HNSCC patients from China often drank liquor with very high concentration of alcohol (usually >50%), whereas Westerners usually consume drinks with much lower concentration of alcohol. Such high concentrations of alcohol may stimulate oral mucosa and destroy bacteria balance, influencing the disease course of advanced HNSCCC [21]. We cannot exclude the possibilities of limited sample size and/or other factors that may have contributed to this observation.

This study was a retrospective case-control study and had some limitations. In the present study, we chose IHC to evaluate XIAP expression instead of some quantitative methods primary because of the unavailability of fresh biopsy tissues. Although IHC is a semi-quantitative method, it is now the most commonly used, simplest and most cost effective protocol in clinical work [22]. Also, the rate of high XIAP expression in the pre-chemotherapy samples was only 20.83%, whereas the chemotherapy response rate (CR+PR) of the patients was 43.34%. It is more than likely that many factors may contribute to the overall drug response in advanced HNSCC; XIAP expression may just be one of many factors involved.

Findings from the current study have potentially important clinical implications. First, our study showed, for the first time that XIAP expression is associated with chemotherapy response and may be used as a biomarker to predict clinical outcomes of advanced HNSCC patients, particularly to those who have had cisplatin-based chemotherapeutic therapy. Second, XIAP expression may be a useful biomarker to select patients who have the greatest chance of benefiting from cisplatin-based neoadjuvant chemotherapy. Finally, the causal relationship between XIAP expression level and chemotherapy response indicate that down-regulation of XIAP might be a promising adjuvant therapy for advanced HNSCC patients.

## Materials and Methods

### Ethics statement

This study was approved by the Ethics Committee of Ninth People's Hospital, Shanghai Jiao Tong University School of Medicine and carried out according to the recommendations of the Declaration of Helsinki. No informed consent (written or verbal) was obtained for use of retrospective tissue samples from the patients within this study, many of whom were deceased, since the Ethics Committee, who waived the need for consent, did not deem this necessary. All samples were anonymous.

### Cell Culture

Human HNSCC cell line CAL27, which was resistant to treatment with cisplatin, was obtained from the American Tissue Cell Collection (Manassas, VA, USA) and cultured in DMEM (Gibco, USA) supplemented with 10% fetal bovine serum (FBS), 1% glutamine, and 1% penicillin-streptomycin. WSU-HN13 cell line[23] was gifted from University of Maryland Dental School (Baltimore, MD, USA) and also cultured in DMEM (Gibco, USA) supplemented with 10% fetal bovine serum (FBS), 1% glutamine, and 1% penicillin-streptomycin.

### Patients and Tumor Specimens

Sixty patients with advanced HNSCC (clinical stage III/Iva; UICC/AJCC. 7 ed., 2010) were recruited in this study. All patients have accepted cisplatin-based neoadjuvant chemotherapy followed by radical tumor resection within two to three weeks of completing chemotherapy at the Department of Oral and Maxillofacial Surgery, Ninth People's Hospital, Shanghai Jiao Tong University from January 1999 to December 2004.

The clinical response of chemotherapy was evaluated no less than 2 weeks after patients completed chemotherapy according to response evaluation criteria in solid tumors (RECIST) [24]. A CR was defined as the complete disappearance of all measurable lesions, without the appearance of any new lesions. A PR was defined as a reduction in bi-dimensionally measurable lesions by at least 50 percent of the sum of the products of their largest perpendicular diameters and an absence of progression in other lesions, without the appearance of any new lesions. SD [9] was defined as a reduction in tumor volume of less than 50 percent or an increase in the volume of one or more measurable lesions of less than 25 percent, without the appearance of any new lesions. PD was defined as an increase in the size of at least one bi-dimensionally measurable lesion by at least 25 percent and the appearance of new lesions. Patients' clinicopathologic information is presented in **Table 4**. All patients were treated with standard curative operations with negative resection margin. All patients received post-operative radiotherapy within two-six weeks of completing surgery. Total dose for primary tumor area and neck of positive nodes was 6000cGy and for primary tumor and neck of negative nodes was 5000 cGy.

**Table 4:** Clinical Characteristics of the patients who participated in study (n = 60).

Characteristics	No. of Patients	%
Gender		
Male	39	65
Female	21	35
Age		
<60	37	62
≥60	23	38
cTNM stage		
III	21	35
IV	39	65
Pathologic grade		
I	31	52
II	25	42
III	4	6
chemoresponse		
SD+PD	26	43
PR+CR	34	57
Smoking history		
Smoker	27	45
Nonsmoker	33	55
Alcohol history		
Drinker	25	42
Nondrinker	35	58
Site		
Tongue	24	40
Gingiva	11	18
Buccal mucosa	12	20
Floor of the mouth	5	8
Oropharynx	4	7
Hard palate	4	7
Nasal sinuses	0	0

### Western Blot Analysis

Total protein was lysed in 2× lysis buffer containing 125 mM Tris-HCl (pH 6.8), 5% w/v SDS, and 24.75% glycerol. 40 µg proteins were separated using 12% SDS-PAGE and then transferred to PVDF membranes. After overnight incubation with monoclonal mouse-anti-human XIAP (BD, USA) in a dilution of 1∶4000 and one hour incubation with IRDye 800CW goat anti-mouse secondary antibody (LI-COR, USA), the signal was scanned and analyzed using the Odyssey Infrared Imaging System (LI-COR Biosciences, USA). β-actin (Sigma-Aldrich, USA) was used as an internal control.

### Real-time PCR

The total RNA was isolated using TRIzol reagent (Invitrogen, USA). 1 µg total RNA wasreverse transcribed into cDNA using oligo-dT primer and PrimeScript II RTase (TaKaRa, Japan) according to the manufacturer's instructions. Real-time PCR was performed with Thermal Cycler Dice Real Time System (TaKaRa, Japan). Primers for PCR were designed with Primer Express® software v3.0 (Applied Biosystem, USA). The primer sequences of XIAP were: 5′-CCGGCTGTCCTGGCGCGAAA-3′ and 5′-GCTCGTGCCAGTGTTGATGCTGA-3′. The primer sequences of β-actin were: 5′-CCTGGCACCCAGCACAAT-3′ and 5′-GGGCCGGACTCGTCATACT-3′, and the primer sequences of GAPDH were: 5′- AATTGAGCCCGCAGCCTCCC -3′ and 5′-ACCAGGCGCCCAATACGACC-3′. All the primers were separated by at least one intron on the corresponding genomic DNA. Dissociation curve analysis was included in all reactions to exclude non-specific amplification. The relative quantity of XIAP mRNA level was calculated based on the standard ΔΔCT methods [25]. Both β-actin and GAPDH were used as internal control.

### siRNA Knockdown

Three anti-XIAP siRNAs each targeting the two splice variants of XIAP and one FAM-labeled negative control siRNA (non-target sequence), were synthesized by GenePharma (Shanghai, China). The sequences were: siRNA1: GGUCAGUACAAAGUUGAAATT, siRNA2: GCAGGUUGUAGAUAUAUCATT, siRNA3: CCGGAAUCUUAAUAUUCGATT and negative control: AAUUCUCCGAACGUGUCACGU. Transfection was performed using Lipofectamine 2000 (Invitrogen, USA) following manufacturer's protocol.

### Immunohistochemistry

Pre- and post-chemotherapy representative tissue paraffin blocks were cut into 5 µm sections for standard immunohistochemical staining (IHC). After heat-induced antigen retrieval in citric acid buffer (pH7.0) for 20 min and blocking in 5% Goat serum for 30 min , slides were incubated with monoclonal mouse anti-human XIAP (BD, USA) at a dilution of 1∶100 at 4°C overnight. The omission of the primary antibody served as negative control. Bound antibody was detected by a Super Sensitive IHC Detection System (BioGenex, USA), according to the manufacturer's protocol. The sections were visualized with diaminobenzidine tetrahydrochloride (Sigma, USA) solution and counterstained with Harris hematoxylin. The staining result was determined by counting 1000 tumor cells in three 100× magnification fields by two independent pathologists and further classified as low expression (the percentage of positive rate <25%) and high expression (the percentage of positive rate ≥25%).

### Drug Sensitivity Assay

Cells were plated in 96-well plates at a density of 4×10^3^ cells/well and further incubated for 24 h. 6 h after transfection with negative control or siRNA, the medium was removed and replaced with fresh medium containingone of serial dilutions of DDP for another 72 h, including a negative control without DDP. Then, 20 µl sterile MTT dye (3-[4,5-dimethylthiazol-2-yl]-2,5-di-phenyltetrazolium bromide, 5 mg dissolved in 1 ml phosphate-buffered saline; Sigma, USA) was added to the culture medium to a final concentration of 0.5 mg/ml and incubated at 37°C for 4 h. Subsequently, the formazan crystals were solubilized with 150 µl of dimethylsulfoxide for 10 min. Spectrometric absorbance at 490 nm was measured with a microplate reader. Each experiment was performed in triplicate.

### Statistical Analysis

The SPSS 17.0 software package was used for statistical analysis. Frequencies were compared with Fisher's exact test, ×2 contingency test, or non-parametric tests as appropriate. We estimated survival and time-to-progression curves using the Kaplan-Meier method and compared them using a two-sided log-rank test. Multiple logistic regressions that used a Cox proportional hazards model were used to determine whether the molecular characteristics of the tumors independently predicted survival in our cohort of advanced HNSCC patients. P<0.05 were considered statistically significant.
